# Responses in early visual areas to contour integration are context dependent

**DOI:** 10.1167/16.8.19

**Published:** 2016-06-30

**Authors:** Cheng Qiu, Philip C. Burton, Daniel Kersten, Cheryl A. Olman

**Affiliations:** qiuxx077@umn.edu; burto108@umn.edu; kersten@umn.edu; caolman@umn.edu; Department of Psychology, University of Minnesota, Minneapolis, MN, USA; Office of the Associate Dean for Research, College of Liberal Arts, University of Minnesota, Minneapolis, MN, USA

**Keywords:** *contour integration*, *fMRI*, *early visual areas*

## Abstract

It has been shown that early visual areas are involved in contour processing. However, it is not clear how local and global context interact to influence responses in those areas, nor has the interarea coordination that yields coherent structural percepts been fully studied, especially in human observers. In this study, we used functional magnetic resonance imaging (fMRI) to measure activity in early visual cortex while observers performed a contour detection task in which alignment of Gabor elements and background clutter were manipulated. Six regions of interest (two regions, containing either the cortex representing the target or the background clutter, in each of areas V1, V2, and V3) were predefined using separate target versus background functional localizer scans. The first analysis using a general linear model showed that in the presence of background clutter, responses in V1 and V2 target regions of interest were significantly stronger to aligned than unaligned contours, whereas when background clutter was absent, no significant difference was observed. The second analysis using interarea correlations showed that with background clutter, there was an increase in V1–V2 coordination within the target regions when perceiving aligned versus unaligned contours; without clutter, however, correlations between V1 and V2 were similar no matter whether aligned contours were present or not. Both the average response magnitude and the connectivity analysis suggest different mechanisms support contour processing with or without background distractors. Coordination between V1 and V2 may play a major role in coherent structure perception, especially with complex scene organization.

## Introduction

Contour integration involves grouping local features across several levels of abstraction and a range of spatial scales. Small, similar elements positioned closely along an invisible smooth path are perceptually organized as due to a continuous contour. This grouping process is enhanced if the elements have orientations that align with the path (Field, Hayes, & Hess, 1993; Hess & Field, 1999; Kovács, 1996; Li & Gilbert, 2002). Further, knowledge of the global form of the path contributes to local integration, such as the form closing (Kovács, 1996; Kovács & Julesz, 1993, 1994) and smoothness (Pettet, 1999; Pettet, McKee, & Grzywacz, 1998), and the global knowledge is often necessary to disambiguate competing local groupings in cluttered scenes (Ullman & Sha'ashua, 1988).

It has been reported repeatedly that cortical areas higher in the visual hierarchy such as the lateral occipital complex (LOC) and inferotemporal cortex (IT) show selectivity to coherent contours (Altmann, Bülthoff, & Kourtzi, 2003; Cardin, Friston, & Zeki, 2010; Dumoulin, Dakin, & Hess, 2008; Dumoulin & Hess, 2006; Kourtzi, Tolias, Altmann, Augath, & Logothetis, 2003; Mendola, Dale, Fischl, Liu, & Tootell, 1999; Murray, Kersten, Olshausen, Schrater, & Woods, 2002; Tanskanen, Saarinen, Parkkonen, & Hari, 2008), which is also consistent with results of shape or object perception from those areas (Fang, Kersten, & Murray, 2008; Grill-Spector, Kourtzi, & Kanwisher, 2001; Grill-Spector, Kushnir, Edelman, Itzchak, & Malach, 1998; Grill-Spector, Kushnir, Hendler et al., 1998; Gross, Rocha-Miranda, & Bender, 1972; Haxby et al., 2001; Kourtzi & Kanwisher, 2000; Logothetis & Sheinberg, 1996; Tanaka, 1996). However, responses to similar contour stimuli in early visual areas are still controversial. While most neurons in the primary visual cortex (V1) are thought to have small receptive fields, it is known that their responses are modulated by contextual information from outside their receptive fields (Allman, Miezin, & McGuinness, 1985; Fitzpatrick, 2000). However, there are substantial conflicting results on how scene context or global perception affects responses in early visual areas, including V1.

Both enhancement (Altmann et al., 2003; Bauer & Heinze, 2002; Chen et al., 2014; Kapadia, Ito, Gilbert, & Westheimer, 1995; Kourtzi et al., 2003; Li, Piëch, & Gilbert, 2006; McManus, Li, & Gilbert, 2011; Roelfsema, Lamme, & Spekreijse, 2004) and suppression (Cardin et al., 2010; Dumoulin & Hess, 2006; Murray et al., 2002; Murray, Schrater, & Kersten, 2004) of cortical responses to coherent contours relative to scrambled elements have been observed in V1 and/or other early visual areas. Recording from individual V1 neurons in monkeys, Kapadia et al. (1995) and Li et al. (2006) showed that multiple randomly placed and oriented line segments outside the neuron's receptive field would inhibit its response to an optimally oriented line within its receptive field; however, once some of the surround segments were placed collinearly with the central line, the response was then facilitated. Similar results have been demonstrated using functional neuroimaging in both monkeys and human subjects (Altmann et al., 2003; Kourtzi et al., 2003): cortical responses from early visual areas including areas V1, V2, and V3 showed selectivity to coherent patterns of closed contours embedded in a field of randomly oriented segments.

In contrast, Cardin et al. (2010) showed lower activity in areas V1/V2 for collinear patterns than for noncollinear ones. Dumoulin and Hess (2006) also showed weaker activity in early visual areas to a 100% coherence circular pattern but stronger activity to scrambled patterns. In a third study, Murray et al. (2002) used line drawings as stimuli and showed smaller responses in V1 to lines that formed two- and three-dimensional shapes than random lines. These were not the only results showing deactivation in early visual areas to stimulus regularities—early visual areas also respond less to coherent than incoherent motion (Händel, Lutzenberger, Thier, & Haarmeier, 2007; Harrison, Stephan, Rees, & Friston, 2007; McKeefry, Watson, Frackowiak, Fong, & Zeki, 1997). A similar trend has been observed with coherent versus scrambled natural images using functional imaging in human observers (Grill-Spector, Kushnir, Hendler et al., 1998; Lerner, Hendler, Ben-Bashat, Harel, & Malach, 2001; Paradis et al., 2000).

We are interested in how the enhancement and suppression of cortical responses in early visual areas to coherent contours could both be true. In either case, neurons in V1 are responding to the same coherent circular contour, but they show different response patterns based on different studies listed above. We think the response pattern may highly depend on stimulus context: results showing response increase mainly used contours embedded in clutter (i.e., with randomly orientated and placed segments in the background), but the ones showing a decrease were usually using isolated structure or if any, with just uniform background. To test this, we designed a 2 × 2 experiment in which context and contour coherence were manipulated, and we used fMRI to record the blood oxygenation level-dependent (BOLD) signal from the retinotopically corresponding regions in early visual areas (V1, V2, and V3). Observers performed a contour detection task in the experiment. When there was no background clutter present, the BOLD responses in regions corresponding to the location of the target were slightly suppressed by aligned contours compared with the unaligned; however, with background clutter, the responses were significantly enhanced by contour alignment. By analyzing the effect of context and contour coherence on correlations between responses in early visual areas, we further demonstrated that the coordination between the retinotopically relevant regions in V1 and V2 was dependent upon the experimental conditions. These results suggest the involvement of multiple strategies in contour integration, and they interact according to the context.

## Materials and methods

### Participants

Fifteen observers (mean age: 29 years old; nine men, six women) with normal or corrected-to-normal visual acuity participated in the study. The observers provided informed written consent under an experimental protocol that was in accordance with safety guidelines for MRI research and was approved by the Institutional Review Board at the University of Minnesota.

### Stimuli

**Figure 1:**
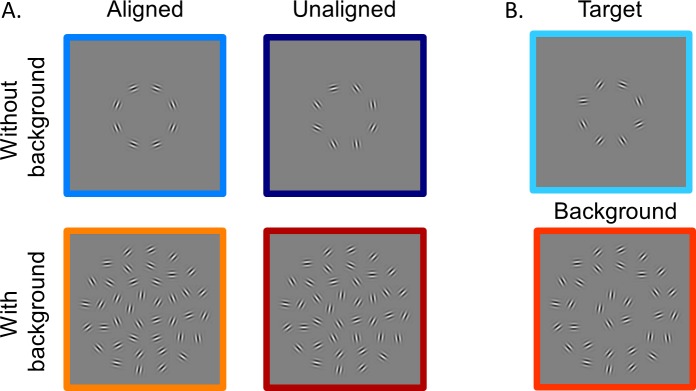
(A) Example stimuli from the four test conditions with a circular contour detection task. (B) Example stimuli from the target (above) versus background (below) differential localizer scan.

Figure 1 provides examples of the stimuli used, which were generated and presented with Matlab (R2010b; MathWorks, Inc., Natick, MA) using the Psychtoolbox extensions (Brainard, 1997; Pelli, 1997). The target region consisted of eight Gabor patches, which were centered at equal intervals along the circumference of an invisible circle centered at fixation with a radius of 2°; that is, these patches were evenly spaced at 2° eccentricity from the fixation. Each Gabor patch consisted of a 4 c/° sinusoidal grating with a random phase offset modulated by a Gaussian envelope with full width at half-maximum of 0.4° (*σ* = 0.17°). In an aligned condition, the grating orientation of each Gabor patch was aligned with the tangent line to the invisible circle at the point of patch center. These Gabor patches therefore could be perceived as forming a complete circle. In an unaligned condition, the grating orientation of each Gabor patch was randomly generated. The background clutter consisting of the same Gabor elements as the target was located along invisible circles centered at fixation and with radii of 1.2°, 2.9°, and 4.0°. Along each circle, the distance between every two Gabor patches was the same as in the target region (1.6°), and the numbers of Gabor patches along each eccentricity were 5, 12, and 16, respectively. The grating orientations of these background patches were randomly generated. All Gabor patches had an 80% Michelson contrast and were presented on a mean gray background. The four experimental conditions included aligned target only, unaligned target only, aligned target with background, and unaligned target with background.

### Functional MRI experiments

Stimuli for the first four observers were presented on a NEC 2190UXi monitor with resolution of 1024 × 768 pixels and a refresh rate of 60 Hz. The monitor had a mean luminance of 110 cd/m^2^. The monitor was mounted to the back wall of the scanning suite; observers viewed the monitor through a mirror mounted to the top of the head coil so that it subtended 12° of visual angle in the horizontal direction and 9° in the vertical direction. For the rest of the observers, stimuli were back-projected via a Sony video projector (spatial resolution of 1024 × 768 pixels, 60 Hz refresh rate, and 120 cd/m^2^ mean luminance) onto a translucent screen placed inside the scanner bore. Observers viewed the stimuli from a distance of 97.5 cm through a mirror located above their eyes (mounted on the head coil), which gave a total image area subtending 26° × 20°.

Functional MRI data were collected using a 3T Siemens Trio scanner (Siemens, Erlangen, Germany) with a 12-channel head array coil. EPI data were acquired with a field of view 128 mm × 256 mm and a matrix size of 64 × 128 for an in-plane resolution of 2 mm × 2 mm. Slice thickness was 2 mm without interslice gap, and number of slices was 20. Echo time (TE) was 30 ms, repetition time (TR) was 1.5 s, and flip angle was 80°. Four out of the 15 datasets were collected in an axial direction, and the other 11 were in a coronal orientation. Both covered the early visual areas V1, V2, and V3.

**Figure 2:**
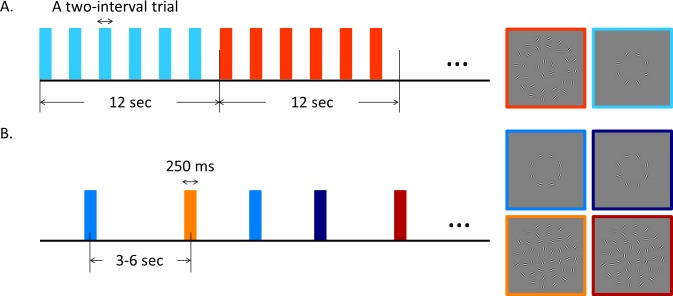
Experimental procedure. (A) An example of block-designed functional localizer scans used to define target or background retinotopically corresponding ROIs. (B) Event-related scans with four experimental conditions.

A scanning session (Figure 2A) contained three runs (runs 1, 5, and 8) of block-design functional localizers and five event-related runs (runs 2, 3, 4, 6, and 7). Observers were instructed to maintain their fixation on a white square at the center while performing behavioral tasks during both localizer and event-related scans. Behavioral responses were recorded using a fiber-optic button box (Current Designs, Philadelphia, PA).

Regions of interest (ROIs) were defined by block-designed functional localizer scans (Figure 2A). Each block lasted 12 s, and each run contained 11 “on” blocks alternating with 10 “off” blocks. Each localizer scan thus lasted 252 s. During “on” blocks, randomly oriented Gabor patches at the target region were presented; during “off” blocks only clutter Gabor patches at the background region were presented. The first half-cycle “on” block was discarded before analysis, and the remaining blocks were alternating in 10 cycles per run. During each block, a two-interval trial occurred every 2 s (six trials per block). Duration for both intervals was 200 ms, which were separated by a 200 ms interstimulus interval (ISI). Observers were instructed to press the button when the stimuli from two intervals were the same (with a probability of 12.5%). The fixation square turned green for a correct response and red otherwise.

The event-related runs (Figure 2B) measured BOLD response to four experimental conditions: aligned target only (alnb, aligned/no background), unaligned target only (uanb, unaligned/no background), aligned target with background (albg), and unaligned target with background (uabg). Stimulus duration was 250 ms, and intertrial intervals (ITIs) were 3, 4.5, or 6 s. ITI was randomly assigned and uniformly distributed. Each run included 20 trials for each condition, thus a total of 80 trials per run. The average run length was about 380 s. Observers were required to press the blue button if they perceived an aligned circle, and press the red button if not. Feedback was provided after each trial.

### Anatomical acquisition and visual area mapping

Prior to the fMRI experiments, each observer participated in a separate retinotopic mapping session, in which a T_1_-weighted anatomical image (MP-RAGE, 1 mm isotropic resolution) was also collected for anatomical reference and cortical surface definition. Gray/white matter segmentation, cortical surface reconstruction, and surface inflation and flattening were completed using FreeSurfer (Dale, Fischl, & Sereno, 1999; Fischl, Sereno, & Dale, 1999). Standard retinotopic mapping including four runs of clockwise/counterclockwise rotating wedges and two runs of expanding/contracting rings (DeYoe et al., 1996; Engel, Glover, & Wandell, 1997; Sereno et al., 1995) was used to identify the early visual areas V1, V2, and V3. Defined visual areas were registered to the reference anatomy for each observer.

### Preprocessing and functional localizers

Functional data was motion corrected using Analysis of Functional NeuroImages software (AFNI; Cox, 1996), with reference to the volume right before a within-session fieldmap image. The motion-corrected data was unwarped using FSL FUGUE to correct distortions introduced by magnetic field inhomogeneities (Smith et al., 2004). High-pass filtering was also applied to the functional localizers data: temporal frequencies below four cycles per run were removed. The preprocessed functional data were then aligned to anatomical reference data using mrAlign implemented in Matlab (MathWorks, Inc.; http://gru.stanford.edu/doku.php/mrTools/overview).

ROIs were defined based on both retinotopic visual areas and functional localizers for each observer. Three repetitions of the functional localizers were averaged to define the ROIs responding to the target (tg) or background (bkgd) regions. For each voxel coherence (unsigned correlation, computed in the Fourier domain as the amplitude of the stimulus-related Fourier component normalized by the square root of the integrated power spectrum) with a sinusoid at the block-alternation frequency, 10 cycles per run, was calculated in the averaged localizer scans (Bandettini, Jesmanowicz, Wong, & Hyde, 1993; Engel et al., 1997). The voxels with coherence exceeding 0.30 were included in the ROIs. The voxels in phase with the target representation were assigned to target ROIs (tgROIs), and the voxels in phase with the background representation were assigned to background ROIs (bkgdROIs). ROIs were initially defined on a flattened cortical surface, where V1, V2, and V3 boundaries could be used to identify the ROIs in different visual areas. Selected voxels were translated to the in-plane space for further refinement to include only contiguous clusters of visually responsive voxels, and the defined ROIs were then exported as a binary mask in the space of the functional data. Six ROIs were defined for each observer: tgV1, tgV2, tgV3, bkgdV1, bkgdV2, and bkgdV3.

### Analysis of the event-related data

#### Functional MRI data analysis

Functional image analysis of the event-related runs was conducted using general linear model (GLM) in AFNI with the function 3dDeconvolve. The BOLD response to individual events from each stimulus condition was modeled using the sum of TENT basis functions in 3dDeconvolve, which is a piecewise linear spline function that estimates an impulse response function. The sum of 13 TENT functions was used to cover the duration of 18 s after the stimulus onset, TENT(0, 18, 13). All models were fit separately to each voxel. For each voxel within the predefined ROIs, 13 amplitudes for each stimulus condition were estimated, which were the time course of the estimate hemodynamic response function (HRF) to each condition at the 13 time points (from 0 to 18 s with the time step of 1.5 s). The mean of the first and last two time points was subtracted from each HRF to ensure that it started from and returned to approximately the same baseline level. Estimates from individual voxels were averaged within each of the 6 ROIs, and BOLD response amplitudes were estimated using the difference between the peak response (reached around 4.5–6 s after stimulus onset) and the baseline response. Our analysis focused on response differences between contour aligned and unaligned conditions, instead of direct comparison between aligned or unaligned contours in clutter versus not, to avoid confound of blood stealing from the background stimulus. Response differences between conditions were assessed using a bootstrapping procedure—resampling the 15 subjects' data with replacement within conditions and calculating differences across 10,000 iterations—and a two-sided permutation test was performed to acquire *p* values (Efron & Tibshirani, 1993). Analysis of variance (ANOVA) was also conducted to compare BOLD response amplitudes among conditions in each predefined ROI, and the ANOVA test assumptions of normality and equal variance were checked using the Lilliefors test (Lilliefors, 1967) and Bartlett's test (Snedecor & Cochran, 1956), respectively.

#### Connectivity analysis

In addition to the estimated response to each stimulus condition, we also wanted to know whether interregional connections among these ROIs depend on the experimental conditions. Two functional connectivity analysis methods were used to answer this question. The first method, psychophysiological interactions (PPI), uses interaction terms created from the dot product of the seed region time series and vectors representing different experimental conditions to explain variance in time series from other cortical regions with a GLM analysis, which is generalized PPI or gPPI, as in McLaren, Ries, Xu, and Johnson (2012) and Cisler, Bush, and Steele (2014). If the estimated beta weights for the interaction regressors are different among experimental conditions, the coordination between the test ROI and the seed ROI depends on the conditions. In the second method, beta series correlations, a beta weight for each experimental trial is estimated using GLM, these beta weights are sorted according to the experimental condition during the trial, and then correlation coefficients are calculated between the beta weights for each condition (Rissman, Gazzaley, & D'Esposito, 2004). If these correlation coefficients vary depending on the conditions, connectivity between the two regions is condition-dependent.

##### Psychophysiological interactions:

**Figure 3:**
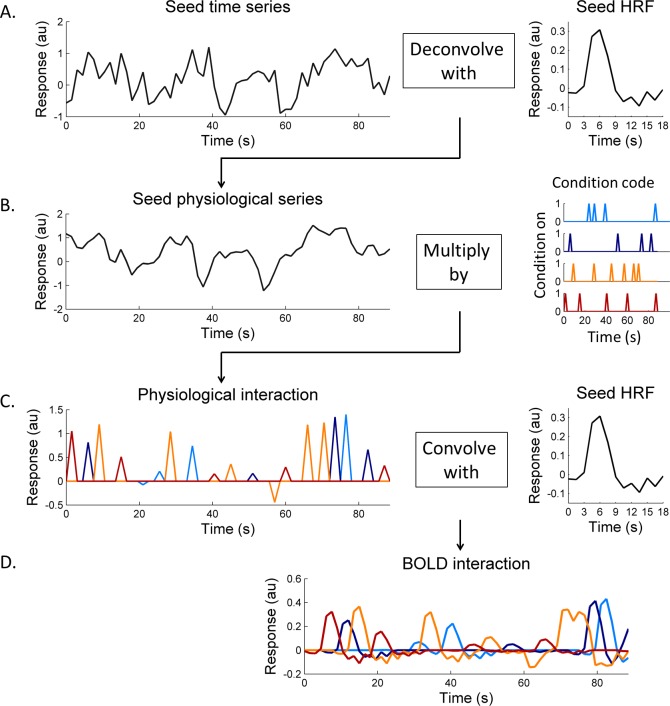
An example of the psychophysiological interactions term. (A) The seed time series were first deconvolved based on the estimated HRF to obtain physiological responses. (B) To combine both the physiological and psychophysical effects we multiplied the seed physiological series by the condition code from each condition separately. (C) The interaction at the physiological level was convolved with the estimated HRF, so we could compare this BOLD level interaction (as shown in D) with residual time series from other ROIs.

First, the stimulus effects modeled by the GLM in the previous analysis were subtracted from the preprocessed event-related dataset to generate a residual dataset for further analysis of the intrinsic interactions between cortical areas. Figure 3 shows an example of the PPI terms, which are used as regressors in a GLM. In order to build the psychophysiological interaction terms, both psychological condition codes (indicating the representation time for a certain stimulus condition) and physiological responses from the seed region were required. According to McLaren et al. (2012) and Cisler et al. (2014), with more than two conditions, separately building one interaction term for each stimulus condition (gPPI) could be more robust to noise and have better model fits than directly using PPI terms with condition contrast (i.e., “1” for one condition while “−1” for a different condition). Therefore, we created one text file matching the length of functional time series for each condition as the condition code (*D_c_*), in which “1” indicated stimulus presentation of that particular condition and “0” for the rest time points (Figure 3B, right panel). The physiological response from the seed region was estimated by deconvolving the time series from the seed region with its HRF estimated from the previous analysis (Figure 3A). Gitelman, Penny, Ashburner, and Friston (2003) and Kim and Horwitz (2008) demonstrated the importance of modeling the underlying neural activity: interaction should be expressed at a neuronal level rather than at the level of hemodynamic responses, with neuronal activity being filtered with an HRF. Interaction was calculated as the product of condition codes and estimated physiological responses (Figure 3B). In order to compare the interaction with BOLD measurements, we then convolved the interaction at a neuronal level with the estimated HRF to obtain a regressor at the level of hemodynamic responses (Figure 3C).

In summary, residual time series from the *i*th ROI, *y_i_*, could be modeled as (Friston et al., 1997):

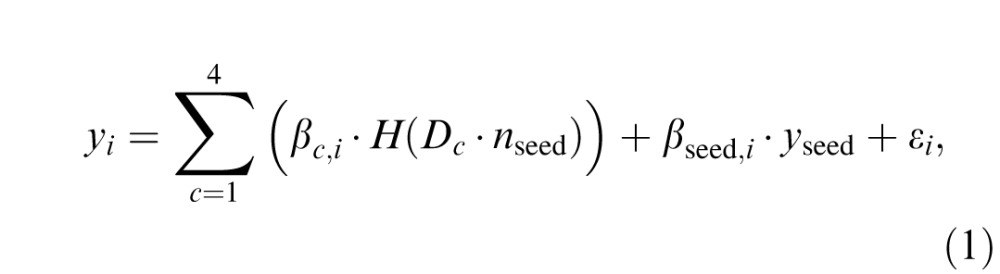
where *c* is the stimulus condition index, here, we have four conditions, *c* = 1, . . . ,4; *β_c_*_,_*_i_* is the correlation coefficient for the PPI regressor of condition *c* at ROI *i*; *β*_seed,_*_i_* is the correlation coefficient for the seed time series regressor at ROI *i*; *H*(·) indicates the convolution operation with the estimated TENT HRF for a certain condition (*c*) in the corresponding region (here, the seed region); *D_c_* is the stimulus presentation code for condition *c*; *y*_seed_ is the time series from the seed region, and *n*_seed_ is the physiological response estimated by deconvolving *y*_seed_ with the estimated HRF; that is, since *y*_seed_ = H(*n*_seed_) + noise, we could solve for *n*_seed_ given the kernel function and *y*_seed_ using deconvolution. Finally, *ε_i_* is an error term at region *i*. The beta estimated for the PPI regressor represents the amount of signal that could be explained by both the response in the seed ROI and the stimulus condition. If the beta estimates at a certain ROI from two conditions are different, the seed may differently influence this ROI between these two conditions. Next, 3dDeconvolve and 3dREMLfit (which estimates and removes noise temporal correlations) were used to estimate coefficients based on the model shown in Equation 1. Estimates of *β_c_*_·_*_i_* from individual voxels were averaged within ROIs, and would be used for assessing statistical significance.

##### Beta series correlations:

With the beta series correlation method (Rissman et al., 2004), we first estimated a beta weight for each experimental trial; that is, modeling the time series from the *i*th ROI, *y_i_*, as

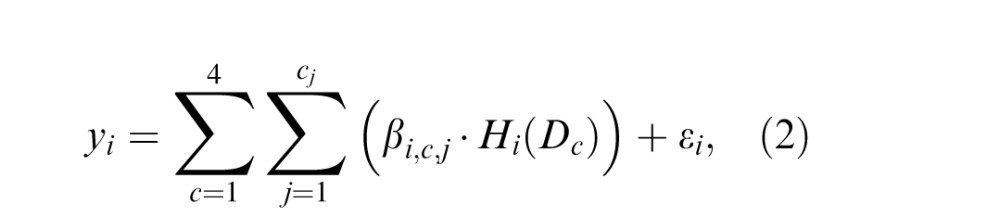
where *c* is the index for the stimulus conditions, *i* is the index for the ROIs, *H_i_*(*D_c_*) indicates the estimated hemodynamic response function at the region *i* for the condition *c*, *β_i_*_,_*_c_*_, _*_j_* is the beta weight for the *i*th ROI during the *j*th trial of the condition *c*, *c_j_* is the total number of trials for the condition *c*, and *ε_i_* is an error term at region *i*. Next, the estimated beta values *β_i_*_,_*_c_*_, _*_j_* ( *j* = 1, . . . ,*c*_*j*_) from the *i*th region were regressed against the *β_i_*_′,_*_c_*_, _*_j_* from the region *i*′ (the seed) according to the conditions, and the correlation coefficient was used to indicate the connectivity between regions *i* and *i*′ under a certain experimental condition.

## Results

**Figure 4:**
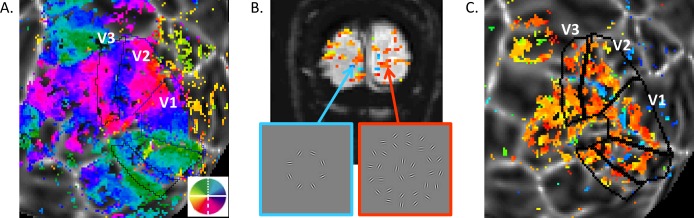
Visual area mapping (A) and functional localizer results (B and C). (A) Angular visual field preference of one observer's left hemisphere obtained from rotating wedge stimulus (overlay on a flattened patch of the cortical surface centered on the occipital pole). The early visual areas are labeled. (B) On one single coronal EPI image, voxels significantly correlated with the block-alternation are color coded based on relative phases—the bluish voxels are in phase with the target presentation, while the orange voxels are in phase with the background stimulus. (C) Data in B was transformed to the flat patch, where a blue target-associated band and two orange background-associated bands could be seen among the early visual areas.

The early visual areas V1, V2, and V3 were manually defined according to the polar angle (Figure 4A) and eccentricity phase maps acquired in separate scanning sessions. Functional localizers (three runs, preprocessed, averaged and subjected to Fourier analysis) were used to define ROIs corresponding to the cortical representations of stimulus target or background regions (Figure 4B, C). On the flat patch, a band of activation associated with the target representation (blue, Figure 4C) and two bands associated with the background representations (orange, Figure 4C) were consistent with the eccentricity features in the early visual areas. Therefore, two sets of ROIs were defined in each visual area corresponding to target (tgROIs) and background regions (bkgdROIs).

### Stimulus-related activity

**Figure 5:**
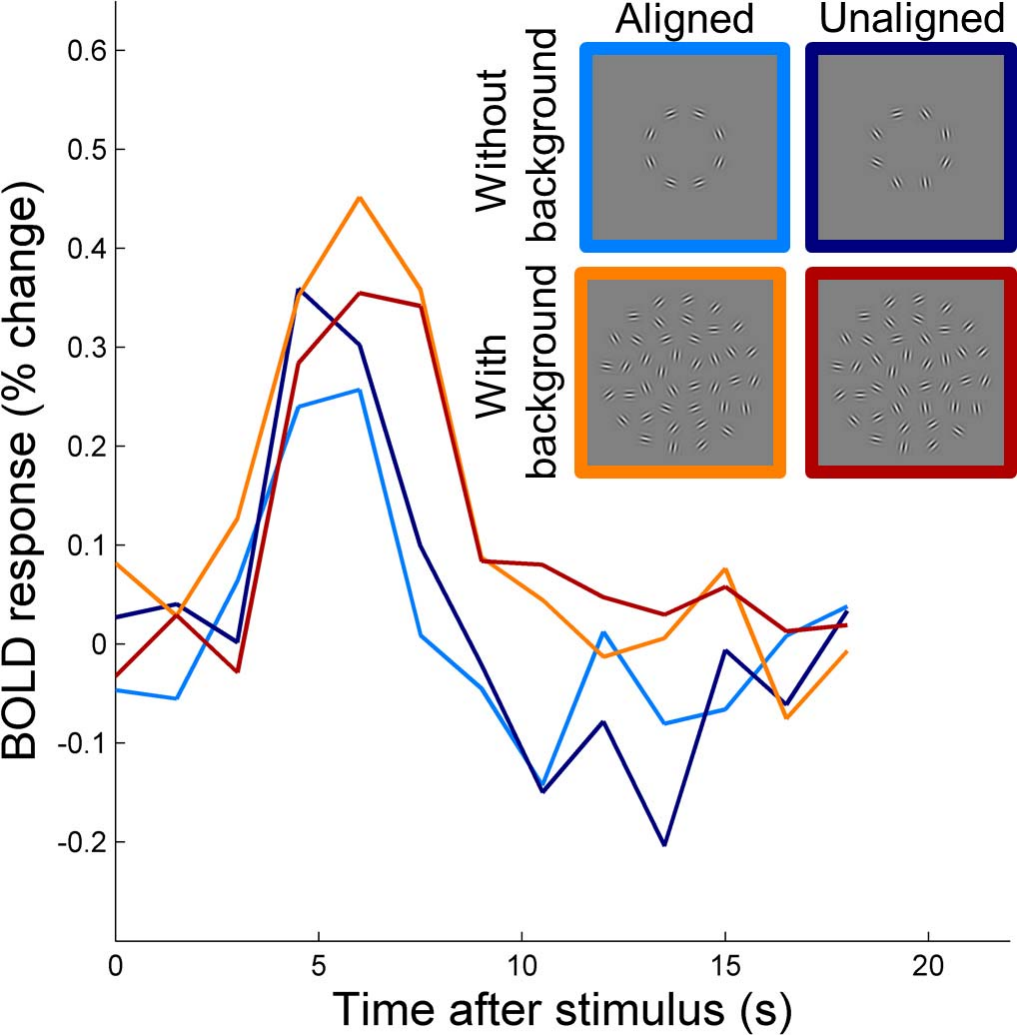
Estimated HRFs from four experimental conditions for one subject's tgV2 ROI. The HRFs were estimated for each voxel, and then averaged within each ROI.

**Figure 6:**
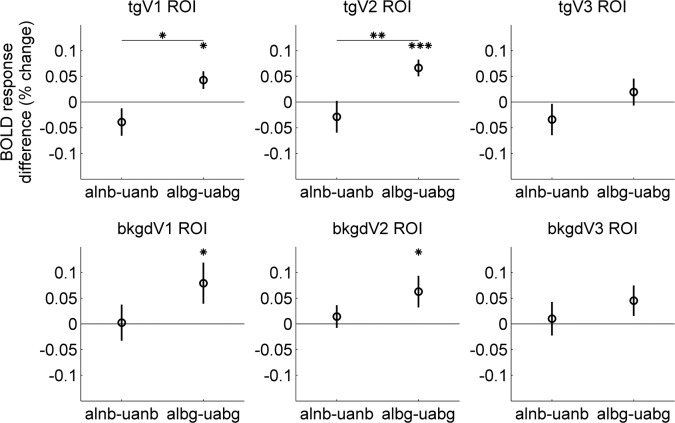
Stimulus-related BOLD response differences among conditions. Each panel shows average data from the 15 observers in one ROI. For each panel, the differences of estimated HRF amplitudes between the aligned and unaligned contours when there was no background are shown on the left, and the differences when there was background clutter are shown on the right. Asterisks indicate statistical significance based on the two-sided permutation test at *p < 0.05, **p < 0.01, and ***p < 0.001. Error bars show ±1 *SE*.

We first looked at the BOLD response magnitude for each experimental condition. Estimated HRFs in one representative subject within the tgV2 ROI are shown in Figure 5. The differences of BOLD responses to the aligned from the unaligned contours for each ROI depend on the background context as shown in Figure 6. When the background was present, the tgV1 and tgV2 ROIs showed a significant preference for the aligned contours (in tgV1, albg − uabg = 0.043 with two-sided permutation test *p* = 0.012, and in tgV2, albg − uabg = 0.066 with two-sided permutation test *p* < 0.001); the bkgdV1 and bkgdV2 ROIs showed a similar trend (in bkgdV1, albg − uabg = 0.079 with two-sided permutation test *p* = 0.028, and in bkgdV2, albg − uabg = 0.063 with two-sided permutation test *p* = 0.019). When there was no background, the responses to aligned contours were weaker than the responses to the unaligned contours in tgV1 (alnb − uanb = −0.039, with two-sided permutation test *p* = 0.13). The tgV3 ROI showed a similar response pattern as in the tgV1 and tgV2 ROIs, but it failed to reach the significance level; this may be related to the simple circular form used in the experiment. Using a two-way ANOVA model (assumptions were satisfied based on the Lilliefors and Bartlett's test) within each visual area (Alignment and Background as fixed effects, and subjects as a random effect), a significant interaction between Alignment and Background was observed in tgV2 ROI, *F*(1, 59) = 7.83, *p* = 0.014, and a similar trend was seen in tgV1 ROI, *F*(1, 59) = 5.85, *p* = 0.030. When we tested for this interaction using a permutation test, tgV2 showed *p* = 0.0062, and tgV1 showed *p* = 0.011. No significant interaction was found in background ROIs. Thus, the regions in V1 and V2 corresponding retinotopically to the target ring responded to the coherent contour differently according to the context.

### Coordination among regions influenced by physiological and psychological states

**Figure 7:**
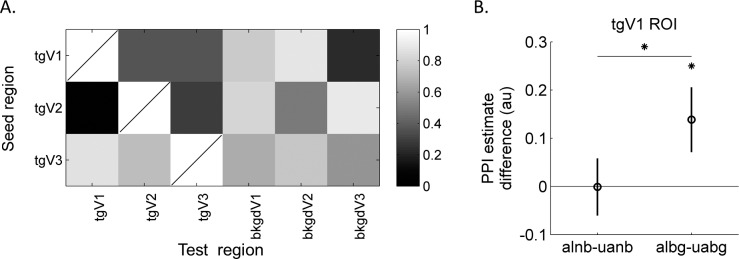
Connectivity results using PPI. (A) The permutation test *p* values for interactions of PPI estimates between the Alignment and Background. A stronger interaction (smaller *p* value) is shown in darker color. Row indicates the seed region. The ROI is not tested on itself. (B) The differences of Beta estimates among conditions in tgV1 are shown when tgV2 was the seed. The mean difference from the 15 observers of estimated beta weights of PPI terms between the aligned and unaligned contours when there was no background is shown on the left, and the difference when there was background clutter was on the right. Error bars show ±1 *SE*. Asterisks show significant levels based on the permutation test at *p < 0.05.

**Figure A1:**
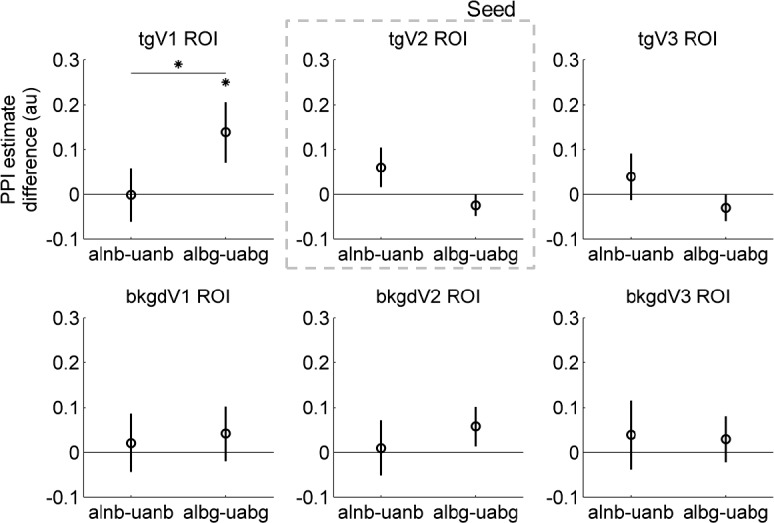
Connectivity results in test ROIs using the PPI analysis when tgV2 was the seed ROI (dashed frame). Each panel shows the average results from the 15 observers in one ROI. Error bars show ±1 *SE*. In tgV1 ROI (the 1^st^ panel), with background clutter (on the right), the aligned condition has larger PPI connectivity estimates than the unaligned condition. The results in other ROIs are not different from zero.

We also explored coordination among defined ROIs using two connectivity analysis methods. In both analyses, the target ROIs were used as the seed regions to assess their effects on other ROIs under various conditions. The background ROIs were not used as the seed, since in half of the conditions, no stimulus was presented (no input visual signal) in background region. The PPI connectivity results are shown in Figure 7. Figure 7A shows the *p* values from permutation tests of PPI estimates for the interaction between the Alignment and Background. One significant interaction was found in tgV1 ROI when tgV2 was used as the seed (with permutation test, *p* = 0.042). In detail, as shown in Figure 7B when the background was present, in tgV1 ROI, beta estimates for the aligned condition were larger than the unaligned (albg − uabg = 0.14, with permutation test *p* = 0.023), but they were not different when the background was absent. Using a two-way ANOVA model (assumptions were satisfied based on the Lilliefors and Bartlett's test) within each ROI (Alignment and Background as fixed effects, and subjects as a random effect), a weak interaction between Alignment and Background was observed in tgV1 ROI, *F*(1, 59) = 3.64, *p* = 0.077. The correlation differences were also retinotopically specific to the target ROIs: no effect was observed in the background ROIs (see Figure A1 for the summary results from other ROIs when tgV2 was the seed).

**Figure 8:**
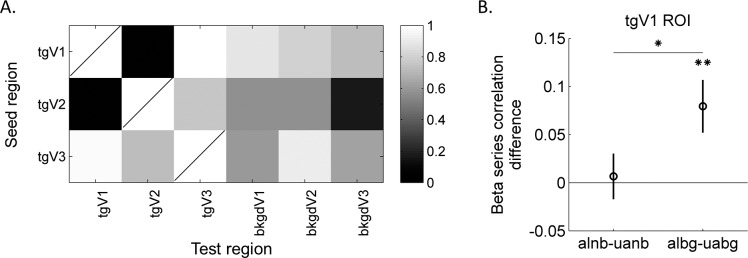
Connectivity results using beta series correlations. (A) The permutation test *p* values for interactions of beta series correlation coefficients between the Alignment and Background. A stronger interaction (small *p* value) is shown in dark color. A significant interaction was observed among tgV1 and tgV2 ROIs. (B) The differences of correlation coefficients among conditions in tgV1 are shown when tgV2 was the seed. The mean difference of beta series correlations from the 15 observers between the aligned and unaligned contours when there was no background is shown on the left, and the difference when there was background clutter was on the right. With background, the aligned condition tended to show larger correlation coefficients between the tgV1 and tgV2 ROIs. Error bars show ±1 *SE*. Asterisks show significant levels based on the permutation test at *p < 0.05 and **p < 0.01.

**Figure A2:**
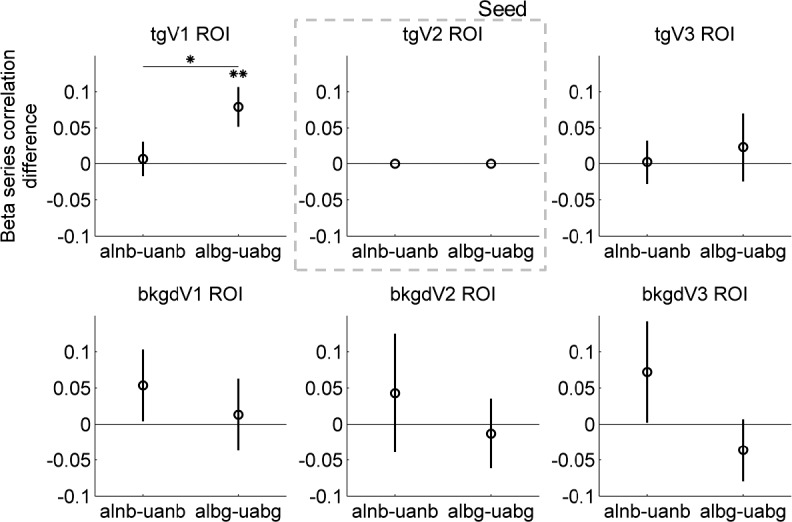
Connectivity results in test ROIs using the beta series method when tgV2 was the seed region (dashed frame). Each panel shows the average results from the 15 observers in one ROI. Error bars show ±1 *SE*. In tgV1 ROI (the 1^st^ panel), with background clutter (on the right), the aligned condition has larger beta series correlation coefficients than the unaligned condition. The results in other ROIs are not different from zero.

With a second interarea connectivity analysis, the beta series correlations, a beta value was first estimated for each experimental trial. Next, these beta values were sorted according to conditions and correlated among ROIs for each condition. The acquired correlation coefficients for each condition and each seed and test ROI pair were tested for interaction between Alignment and Background factors (the permutation test *p* values as shown in Figure 8A). We found that, similar to the PPI results, the interaction was significant in tgV1 when tgV2 was the seed, with permutation test *p* = 0.021 and with ANOVA test, *F*(1, 59) = 4.35, *p* = 0.056. Figure 8B shows differences of beta series correlations estimated in tgV1 against tgV2 ROI (see Figure A2 for results in other ROIs). With the background, the correlation coefficients of beta values from the tgV1 when tgV2 was the seed were larger when the Gabors were aligned (albg − uabg = 0.079, with two-sided permutation test *p* = 0.0068), but no significant difference was observed without background clutter. Due to symmetry of the analysis, the interaction in tgV2 when tgV1 was the seed was also significant. The beta series correlations showed similar sensitivity to tgV1–tgV2 connectivity as using the PPI analysis.

## Discussion

### Response enhancement in early visual areas is context dependent

We investigated how context and contour coherence affect the magnitude and interarea correlations of the fMRI BOLD signal in human early visual areas. The results from our first analysis showed that with clutter in the background, fMRI responses in target ROIs in early visual areas V1 and V2 were larger for aligned than for unaligned contours (Altmann et al., 2003; Kourtzi et al., 2003); while with isolated structure, the responses in V1 target ROIs were slightly larger for the unaligned than for aligned contours (Murray et al., 2002). This interaction between the contour alignment and the background context was significant in the regions retinotopically corresponding to the target stimulation in areas V1 and V2. Area V2 showed the most significant effect, which agrees with its role in extracting features from complex visual scenes (Boynton & Hegdé, 2004; Huang, Hess, & Dakin, 2006; Ito & Komatsu, 2004; Merigan, Nealey, & Maunsell, 1993; Roe, 2003; von der Heydt, Peterhans, & Baumgartner, 1984). In contrast to previous functional imaging studies, we used a separate functional localizer to define the cortical region retinotopically associated with the target contour; we demonstrated that the effect was specific to the target related region, since no significant interaction was observed in the background ROIs.

Our results are consistent with the electrophysiological results reported in Li et al. (2006). They showed a close correlation between the responses of monkey V1 neurons and the perceptual saliency of contours, which was modulated by number of collinear elements or relative spacing between them; however, the correlation could be either positive or negative, depending on the context beyond the collinear elements. Specifically, they found that without the background clutter, neurons in V1 showed facilitation to three collinear lines compared with a single line in their receptive fields, but responses in V1 neurons were inhibited when there were more than three aligned elements. Conversely, with the background clutter, neuronal responses increased monotonically with increasing aligned line segments.

Similarly, we found that the increased response to aligned contours in the target ROIs relied on the presence of background clutter. This facilitative results to aligned contours observed in the presence of the background could be predicted based on flank facilitation on local segments along the contour. Both psychophysics and electrophysiology have shown that when surround segments are positioned within a certain range outside the neuron's receptive field and placed collinearly with the central stimulus, the neuronal responses to the center can be facilitated (Chen & Tyler, 2008; Kapadia et al., 1995; Kapadia, Westheimer, & Gilbert, 2000; Polat & Sagi, 1993, 1994). One interpretation is that the intrinsic horizontal connections in V1 can link neurons with nonoverlapping receptive fields but with similar orientation preference to integrate information over a relatively large visual field (Angelucci et al., 2002; Bosking, Zhang, Schofield, & Fitzpatrick, 1997; Gilbert, 1992; Gilbert & Wiesel, 1989; Li, 1998; Malach, Amir, Harel, & Grinvald, 1993; McGuire, Gilbert, Rivlin, & Wiesel, 1991; Rockland & Lund, 1983; Stettler, Das, Bennett, & Gilbert, 2002; Ts'o, Gilbert, & Wiesel, 1986). Therefore, the facilitated single neuron responses can be associated to form a coherent contour (Field et al., 1993; Hess & Field, 1999; Li & Gilbert, 2002), and cause response increases along the path. Furthermore, feedback signals from higher cortical areas, such as area V4, can also enhance the global contour signals in early visual areas (Chen et al., 2014).

In contrast, the nonfacilitative results may rely more on an understanding of the global scene. The visual system efficiently represents structures that follow natural scene statistics (Attneave, 1954; Barlow, 1961; Geisler, Perry, Super, & Gallogly, 2001; Simoncelli & Olshausen, 2001). Below, we discuss two ways the visual system might achieve this efficiency: predictive coding and disambiguation.

One way to achieve efficiency is to generate and feed back high-level “summary” templates for probable forms of the natural inputs, such as circular forms (Sigman, Cecchi, Gilbert, & Magnasco, 2001), and only signals representing deviations from the predicted templates are subsequently carried forward to be resolved by further processing, a theory referred to as “predictive coding” (Friston, 2005; MacKay, 1956; Mumford, 1992; Murray et al., 2004; Rao & Ballard, 1999). The degree of deviation from the template would be reflected in the magnitude of the *localized* neural activity (but see de-Wit, Kubilius, Wagemans, & Op de Beeck [2012]). In our experiment, predictive coding theory would result in larger responses to the unaligned contour because the elements deviate from the circular template.

Alternatively, the predictions from a higher level could disambiguate the lower-level representation by attenuating responses to unmatched incoming features (Murray et al., 2004; Yuille & Kersten, 2006). For example, the signal for background clutter could be suppressed once the circular foreground structure is detected, and this may decrease overall spatially averaged responses to coherent structure, perhaps for the purpose of metabolic efficiency (Barlow, 1959, 1961). Based on this model, when a coherent target appears, cortical responses to the target would be enhanced whereas responses to surrounding noise would be suppressed, as shown by Gilad et al. (2013) using voltage-sensitive dye imaging in V1 of monkeys.

However, based on our results, the response dependence on the background clutter was only observed at the regions specifically responding to the target ring and no suppression was observed in background ROIs, which are not consistent with the disambiguation theory described above. Although not statistically significant, the enhanced responses in V1 to unaligned contours (in the absence of background clutter) are consistent with the predictive coding idea.

### Coordination between areas V1 and V2

Furthermore, using the connectivity analyses—the psychophysiological interactions and beta series correlations—we found that the coordination between target V1 and target V2 ROIs was also highly dependent on the stimulus conditions. When the contours were presented together with the background clutter, a larger connectivity was observed between tgV1 and tgV2 ROIs for aligned contours than for unaligned ones. However, no connectivity difference was observed when the contours were presented without the background.

Close connections between areas V1 and V2 are well established from anatomical and physiological studies and can be grouped into three categories: feedforward, feedback, and common inputs. In the feedforward category, a large percentage of the cortical inputs in visual area V2 is from area V1 (Felleman & Van Essen, 1991; Sincich, Adams, & Horton, 2003), and the connections form multiple parallel pathways each carrying specific local representations such as color, form, and motion from V1 neurons (Federer et al., 2009; Livingstone & Hubel, 1988; Sincich & Horton, 2002, 2005). Area V1 also receives numerous feedback projections from V2 (Anderson & Martin, 2009; Angelucci et al., 2002; Barone, Batardiere, Knoblauch, & Kennedy, 2000; Girard, Hupé, & Bullier, 2001; Rockland & Virga, 1989; Stettler et al., 2002), but their function could be highly dependent on the visual stimulus (Anderson & Martin, 2009). For example, with one isolated stimulus no change in orientation selectivity was observed in area V1 when area V2 was cooled (Sandell & Schiller, 1982), and only a few V1 neurons were affected by V2 inactivation when simple center-surround stimuli were used (Bullier, Hupé, James, & Girard, 1996; Hupé, James, Girard, & Bullier, 2001). However, feedback may play a stronger role in more complex scenes. For example, given illusory contours induced by abutting gratings, there is evidence that area V2 modulates the orientation representation map in area V1 to provide a signature for a “higher order” contour (Ramsden, Hung, & Roe, 2001; Roe, 2003). The third category of connections that would relate responses in area V1 to V2 is the common inputs to these two areas from the same cortical and subcortical structures (Kennedy & Bullier, 1985). One example is that responses in areas V1 and V2 both are strongly influenced by feedback from area MT (Dakin, 2009; Hupé et al., 1998; Sillito, Cudeiro, & Jones, 2006).

Although the precise mechanism is unclear, the modulation of interarea correlations (aligned vs. unaligned) that we observed between V1 and V2, which were unique to the cluttered conditions, may reflect the concurrent signal transferring required to isolate the target contour from background noise. Similar interarea correlation increases with increasing levels of elements alignment or structure detectability have been observed in previous studies (Cardin et al., 2010; Freeman, Donner, & Heeger, 2011).

### Flexible deployment of local versus global strategies balances accuracy and efficiency

**Figure 9:**
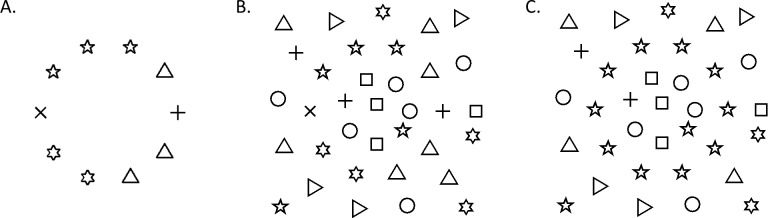
(A) A circular shape could be grouped based on global features. (B) The same circle is not easily identified when surrounded by clutter. Local linkage cues are required. For example, in (C), the nearby pentagrams serve as local cues for the circular shape to be grouped.

Form information can be grouped based on either global or local features. Strategies based on global features (e.g., “elements form a circle”) may rely more on top-down templates: as shown in Figure 9A, a coherent circular shape could be perceived even when individual elements do not all share similar features. In contrast, a local feature-based strategy strongly depends on bottom-up relays, and it relies on similarities among nearby elements (Figure 9C); among clutter, when the nearby elements forming the circle do not share features, local integration processes could fail (Figure 9B; see also Keeble & Hess [1999], Levi & Klein [2000]). In fact, the prediction of human performance for detecting naturalistic contours among background distractors is fairly accurate by applying local grouping functions (Geisler et al., 2001). However, even when the local strategy is dominant, in order to compensate for the potential ambiguities of a merely bottom-up process, a higher-level global template can also be useful (Elder, Krupnik, & Johnston, 2003; Epshtein, Lifshitz, & Ullman, 2008). In all, applying both local and global strategies can be crucial to accurately represent the contour information (Friston, 2005; Kersten, Mamassian, & Yuille, 2004; Kersten & Yuille, 2014).

Further, the use of a global versus local strategy should be dynamically adjusted given the signal-to-noise ratio in the scene in order to improve efficiency in representations (Zhaoping, 2014). For example, in the absence of background clutter, once the system figures out the representation of a coherent circular contour, no signal enhancement of the local alignment is necessary, which may explain the lack of facilitative responses to the coherent target in our study. Overall, balancing the above two strategies according to a greater stimulus context could improve both accuracy and efficiency of the cortical function.

## Conclusion

In summary, we have shown that the cortical responses of human early visual areas to coherent contours are affected by a larger context around them. A locally coherent target enhances neuronal responses to indicate certainty, but this may not be efficient especially when the target could be easily abstracted or explained globally. On the other hand, a system that only relies on the global approach and entirely discards basic representation signals from lower level areas is inflexible and may encounter problems later on, for example, when further operations on other detailed features are required. Therefore, the visual system should be able to apply both local and global strategies and to weight them according to context, such as scene complexity or task difficulty. With the current task, detecting circular contours among simple or complex scenes, we have found that early visual areas V1 and V2 may play an important role in manipulating contour integration strategies under various conditions.

## Supplementary Material
