# Supplementary figures and images for: Responses in early visual areas to contour integration are context dependent
Source: J Vis. 2016 Jun 30;16(8):19. doi: 10.1167/16.8.19 (PMC4946811; doi:10.1167/16.8.19)

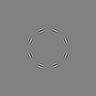

Supplement: Supplementary file 1 [file i1534-7362-16-8-19-icon.gif]
